# Manipulating Heat Shock Factor-1 in *Xenopus* Tadpoles: Neuronal Tissues Are Refractory to Exogenous Expression

**DOI:** 10.1371/journal.pone.0010158

**Published:** 2010-04-13

**Authors:** Ron P. Dirks, Remon van Geel, Sanne M. M. Hensen, Siebe T. van Genesen, Nicolette H. Lubsen

**Affiliations:** Department of Biomolecular Chemistry, Radboud University Nijmegen, Nijmegen, The Netherlands; University of Kent, United Kingdom

## Abstract

**Background:**

The aging related decline of heat shock factor-1 (HSF1) signaling may be causally related to protein aggregation diseases. To model such disease, we tried to cripple HSF1 signaling in the *Xenopus* tadpole.

**Results:**

Over-expression of heat shock factor binding protein-1 did not inhibit the heat shock response in *Xenopus*. RNAi against HSF1 mRNA inhibited the heat shock response by 70% in *Xenopus* A6 cells, but failed in transgenic tadpoles. Expression of XHSF380, a dominant-negative HSF1 mutant, was embryonic lethal, which could be circumvented by delaying expression via a tetracycline inducible promoter. HSF1 signaling is thus essential for embryonic *Xenopus* development. Surprisingly, transgenic expression of the XHSF380 or of full length HSF1, whether driven by a ubiquitous or a neural specific promoter, was not detectable in the larval brain.

**Conclusions:**

Our finding that the majority of neurons, which have little endogenous HSF1, refused to accept transgene-driven expression of HSF1 or its mutant suggests that HSF1 levels are strictly controlled in neuronal tissue.

## Introduction

In healthy cells, accumulation of abnormally folded proteins in the cytoplasm results in the activation of a stress response system, the heat shock response (HSR). The HSR is essential for maintaining proteostasis. Mouse knockout models have shown that heat shock transcription factor-1 (HSF1) is the key regulator of the HSR [1]–[3]. Under normal physiological conditions, HSF1 is thought to exist as part of an inactive hetero-complex that also includes Hsp90, p23 and immunophilin. Exposure of cells to various stressors results in trimerization and hyperphosphorylation of HSF1, followed by binding of the active trimer to heat shock elements in the promoters of heat shock protein genes and subsequent transcription activation (reviewed by [4]). The expression level and thermostability of HSF1, as well as its affinity for heat shock elements are significantly decreased in aged cells compared with young cells, resulting in low efficiency of the HSR ([5]). As the HSR is already poorly developed in healthy neurons, these cells are particularly vulnerable to damage resulting from decreased activity of HSF1 (reviewed by [6]). Restoring the activity of HSF1 or bypassing the crippled HSF1 may protect the aging cell against toxic protein aggregates ([7], [8]; see also [9], [10]). High throughput screens have already resulted in novel compounds that directly or indirectly affect the HSR in cell culture systems (reviewed by [11]).

A number of *in vivo* model systems have been described in which the role of a failing HSR in the etiology of neurological diseases can be studied. Mice carrying null mutant HSF1 genes or expressing a dominant-negative HSF1 are attractive model systems, because the mouse brain closely resembles the human brain; however, small offspring and high costs make rodents less attractive for large scale studies. Invertebrates, such as *C. elegans* and *Drosophila*, are particularly suitable for high throughput experiments, because they have a large offspring and can be easily manipulated. Unfortunately, their nervous system differs considerably from the human brain. Simple vertebrates, such as *Xenopus* and *Danio*, are attractive *in vivo* model systems to study many aspects of human neurological diseases. In addition to the large offspring and low maintenance costs, the basic anatomy of the fish and amphibian brain is highly similar to that of the mammalian brain. Furthermore, tadpoles and zebrafish are translucent, allowing live image analysis of fluorescently labeled proteins. Microinjection of antisense oligonucleotides has already been used to transiently inhibit the expression of HSF1 in zebrafish. This resulted in increased heat shock-induced apoptosis [12], reduced basal expression levels of Hsp70 and abnormal eye development [13].

To be able to study the long term effects of decreased HSF1 activity in neurons and perform high throughput experiments, it would be desirable to develop a model in which the expression of HSF1 is stably inhibited. In this study, we examined if we could mimic the aging-associated decline of the HSR in a stable manner by crippling endogenous HSF1 in *Xenopus* tadpoles. Since a technique for targeted mutagenesis of endogenous genes in *Xenopus laevis* is not (yet) available, we tried to manipulate *Xenopus* HSF1 via three alternative strategies: over expression of heat shock factor binding protein-1 (HSBP1), reported to be a natural inhibitor of HSF1 [14], over expression of a dominant-negative mutant of HSF1, and stable transgene-driven RNA interference (RNAi) directed against HSF1 mRNA. Our surprising finding is that Xenopus tadpole brain is largely refractory to exogenous expression of HSF1.

## Results

### *Xenopus* heat shock factor binding protein-1 is not an efficient inhibitor of the heat shock response

**Figure 1:**
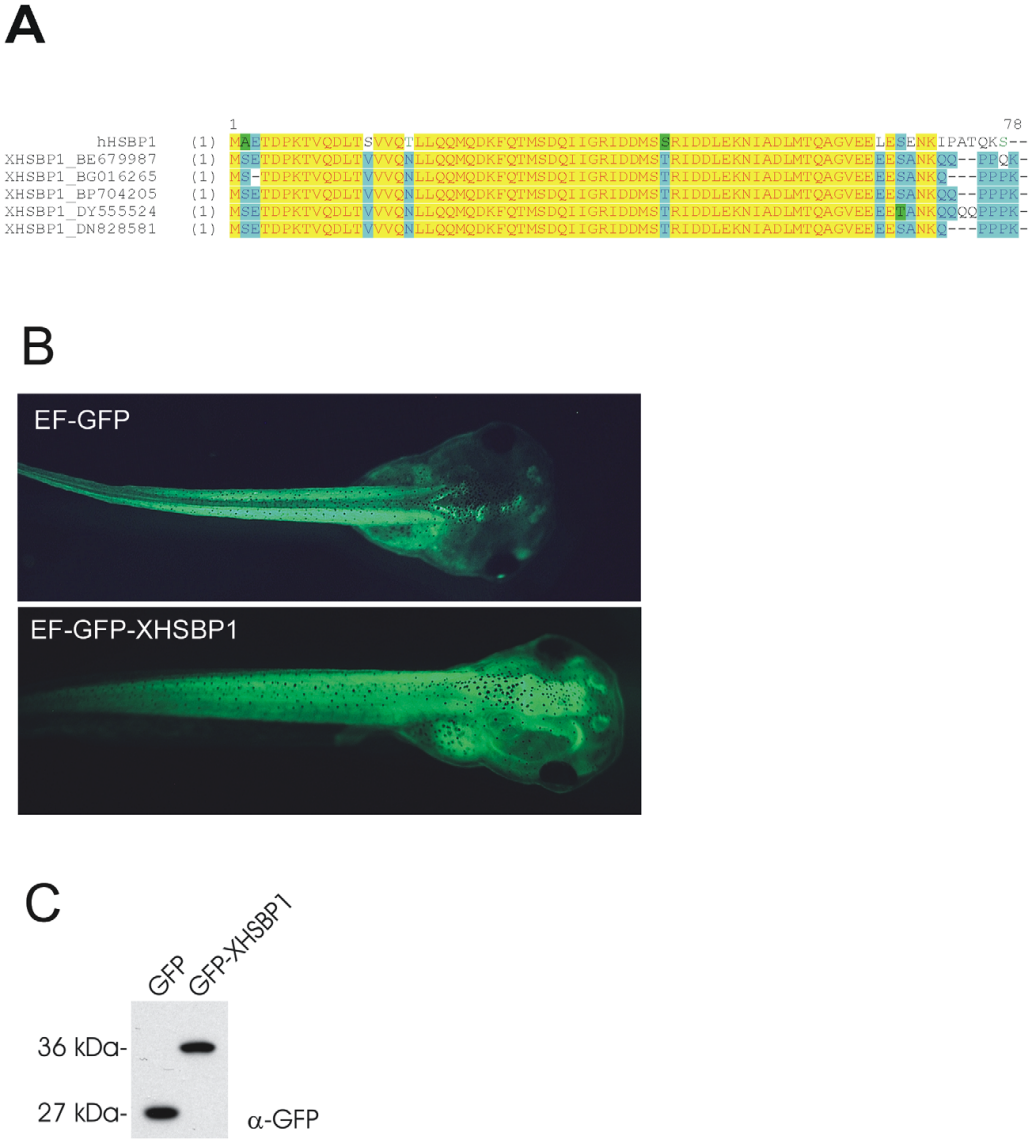
Ubiquitous transgene-driven expression of XHSBP1 in *Xenopus* tadpoles. (**A**) ClustalW alignment of human HSBP1 (top) and HSBP1 sequences deduced from five different *Xenopus laevis* ESTs. The central coiled coil region is highly conserved between man and *Xenopus*. (**B**) Fluorescence microscope analysis of tadpoles carrying the indicated transgenes. Tadpoles with ubiquitous, high level expression of GFP-HSBP1 look normal. (**C**) Western blot analysis of lysates from whole tadpoles carrying the indicated transgenes. Proteins were separated by SDS-PAGE and analyzed with an anti-GFP antibody.

**Figure 2:**
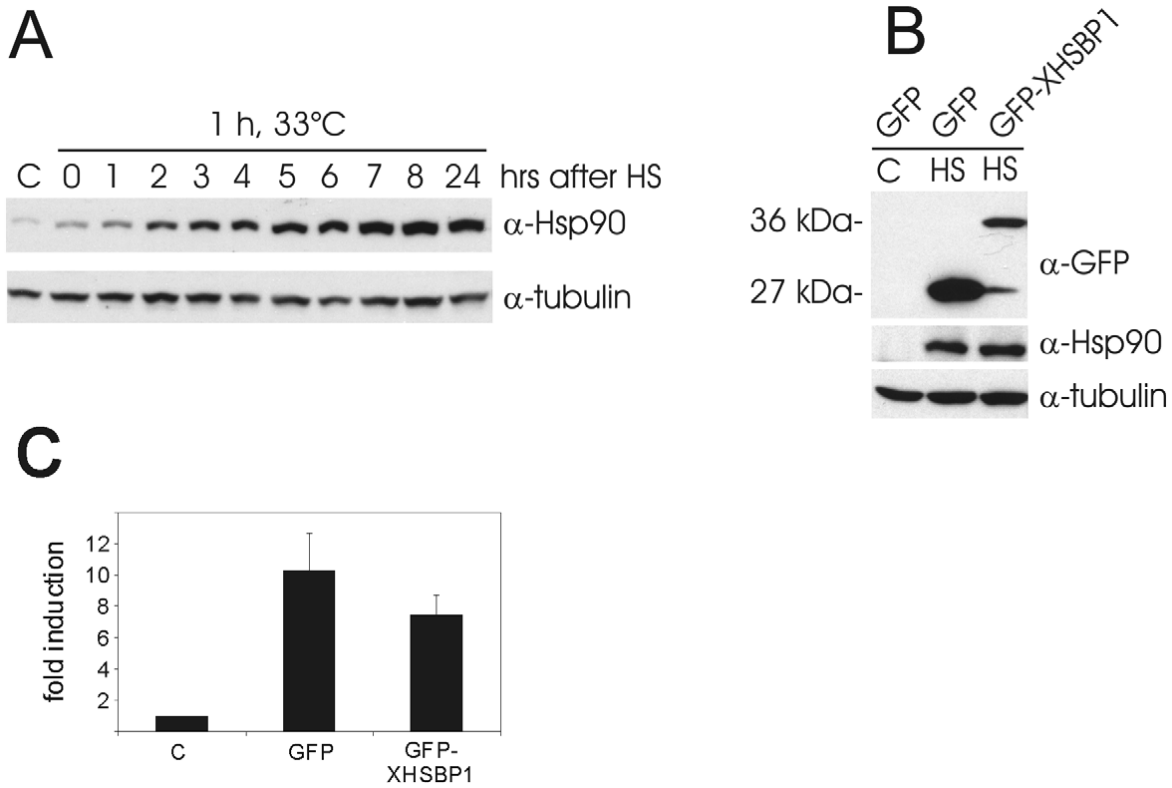
The effect of exogenous expression of GFP-XHSBP1 on the HSR. (**A**) Heat shock induced expression of Hsp90. *Xenopus* A6 kidney epithelial cells were continuously cultured at room temperature (C) or exposed to a 1 h heat shock (33°C) and then cultured at room temperature for the indicated times. Cell lysates were subjected to SDS-PAGE and western blot analysis using anti-Hsp90 and anti-tubulin antibodies. (**B**) Western blot analysis of Hsp90 levels in tadpoles carrying the indicated transgenes. Lysates were made from whole tadpoles before heat shock (C) or after a 1 h heat shock at 33°C followed by 6 h recovery at room temperature (HS). The lysates were subjected to SDS-PAGE and western blot analysis using anti-GFP, anti-Hsp90 and anti-tubulin antibodies. (**C**) Reporter gene analysis of the effect of GFP-XHSBP1 on the HSR. A6 cells were transfected with mixtures of an Hsp70-luciferase reporter, a CMV-β-galactosidase reporter and the indicated plasmids. At 48 h after transfection, cells were exposed to a 1 h heat shock at 33°C. Control cells were left at room temperature. Cell lysates were made at 6 h after heat shock and used for reporter gene assays. Hsp70 promoter activities were determined by dividing luciferase values by the corresponding β-galactosidase values to correct for varying transfection efficiencies. The HSR is indicated as fold induction relative to the activity of the Hsp70 promoter in control cells, which was set at 1 (C). The results are the average of three independent transfections (standard deviations are indicated by error bars).

HSBP1 is a ∼9-kDa polypeptide that was shown to act as a negative regulator of the HSR in cultured mammalian cells and in *C. elegans in vivo* [14]. Since HSBP1 is highly conserved throughout the animal kingdom [15], we examined whether transgene-driven over expression of XHSBP1 could be used to stably inhibit the HSR in *Xenopus* tadpoles. ClustalW alignment of the open reading frames of multiple *Xenopus laevis* HSBP1 ESTs revealed slight variation in the C-terminal part of the protein. However, the central coiled coil region thought to be important for the inhibitory interaction between HSBP1 and HSF1 [15] is identical in all ESTs and nearly identical to that of the human protein (Fig. 1A). The code for XHSBP1 was PCR amplified from adult *Xenopus* brain cDNA and, to allow for quick identification of transgenic tadpoles, fused to the 3′ end of the code for GFP. Tadpoles with ubiquitous expression of the GFP-HSBP1 protein developed normally (Fig. 1B,C). To test whether over expression of GFP-XHSBP1 inhibited the HSR in tadpoles, the endogenous Hsp90 level was monitored by western blot analysis. Basic Hsp90 levels are low in tadpoles as well as in *Xenopus* A6 kidney epithelial cells. Upon heat shock (1 h, 33°C) the level significantly increases, reaching a maximum after approximately 8 h (Fig. 2A,B). The heat shock-induced Hsp90 level was not significantly different between tadpoles expressing GFP-XHSBP1 and non-transgenic controls (Fig 2B), indicating that GFP-XHSBP1 does not inhibit the activity of HSF1. Subsequently, we monitored the effect of GFP-XHSBP1 on an Hsp70 promoter-luciferase reporter plasmid in transiently transfected A6 cells. Upon heat shock, the activity of the Hsp70 promoter increased tenfold in the presence of GFP alone. Co-expression of GFP-XHSBP1 resulted in only 27% reduction of the activity of the Hsp70 promoter (Fig. 2C), which is much less than the 80% reduction observed by others [14].

**Figure 3:**
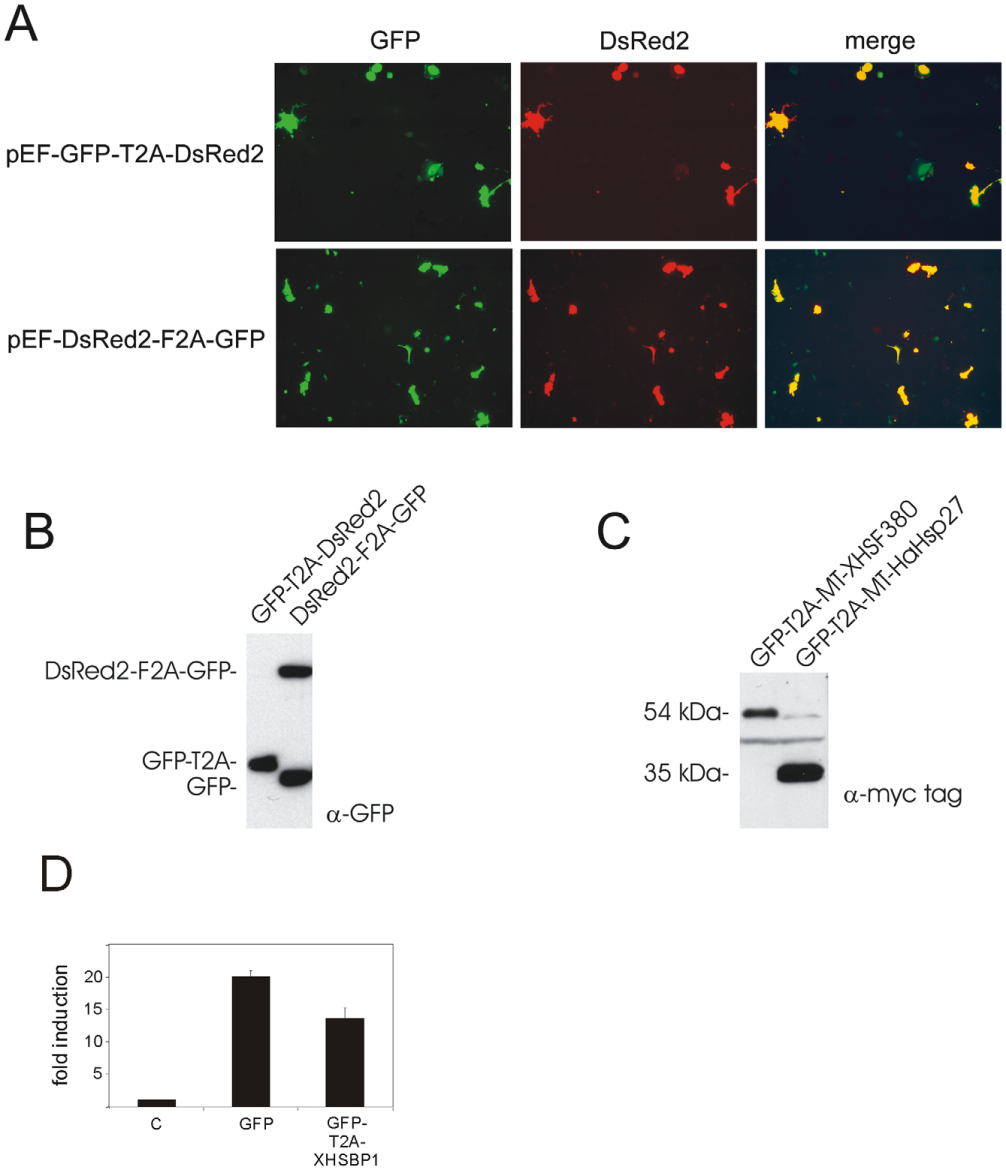
The effect of exogenous expression of XHSBP1 on the HSR. (**A**) Fluorescence microscope analysis of reporter gene expression from bicistronic plasmids. A6 cells were transfected with the indicated dual reporter gene plasmids based on viral 2A peptides. At 24 h after transfection, GFP and DsRed expression was determined by fluorescence microscope analysis. (**B**) Western blot analysis of gene expression from bicistronic plasmids. A6 cells were transfected with the indicated 2A peptide-based plasmids. At 24 h after transfection, cell lysates were made and subjected to SDS-PAGE and western blot analysis using anti-GFP antibodies. (**C**) Western blot analysis of gene expression from bicistronic plasmids. A6 cells were transfected with the indicated 2A peptide-based plasmids. At 24 h after transfection, cell lysates were made and subjected to SDS-PAGE and western blot analysis using anti-GFP and anti-myc tag antibodies. (**D**) Reporter gene analysis of the effect of native XHSBP1 on the HSR. A6 cells were transfected with mixtures of an Hsp70-luciferase reporter, a CMV-β-galactosidase reporter and the indicated plasmids. Relative luciferase activities and -fold induction were determined as described in the legend to fig. 2C. The results are the average of three independent transfections (standard deviations are indicated by error bars).

HSBP1 is a nuclear protein and the size of the GFP tag might prevent proper transport of the fusion protein into the nucleus. In addition, the GFP tag could cause steric hindrance and thereby interfere with the interaction between XHSBP1 and HSF1. Therefore we sought ways to simultaneously express GFP and native XHSBP1 in tadpoles. Since co-expression from an internal ribosome entry site or from tandemly linked transgenes is unreliable and inefficient in transgenic tadpoles (our unpublished observations), we tested whether a ribosome skip system based on viral 2A peptides ([16]) could be used to simultaneously express two proteins in *Xenopus*. Insertion of the code for either the T2A or F2A peptide between the GFP and DsRed2 open reading frames resulted in ∼100% co-fluorescence in A6 cells in both cases (Fig. 3A); however, western blot analysis indicated that ribosomal skipping from the T2A-based bicistronic expression cassette was almost ∼100% efficient, whereas the F2A peptide resulted in only ∼50% skipping efficiency (Fig. 3B). A control experiment, in which GFP and myc-tagged versions of Hsp27 or dominant-negative HSF1 are expressed from a bicistronic transcription unit further demonstrated that the second part of the T2A-based plasmids is also properly expressed (Fig. 3C). The T2A-based system was then used to test the effect of native HSBP1 on co-transfected Hsp70-luciferase reporter in A6 cells. The activity of the Hsp70 promoter was inhibited, but by only ∼30% in the presence of native XHSBP1 (Fig. 3D). In summary, our results indicate that over expression of XHSBP1 is not a successful strategy to inhibit the HSR of *Xenopus*.

### RNAi against HSF1 inhibits HSR in A6 cells but is not effective in tadpoles

As an alternative strategy, we tried to reduce the expression of HSF1 by means of stable, transgene-driven RNAi. Earlier efforts to stably inhibit gene expression via RNAi in *Xenopus* tadpoles were only partially successful. Whereas expression of exogenous GFP could be inhibited by co-expressing long GFP dsRNA from RNA polymerase II promoters [17] or GFP shRNAs from an RNA polymerase III promoter [18], neither polymerase II nor polymerase III promoter-based inverted repeat constructs resulted in stable inhibition of endogenous target genes [17]. We already showed that the human H1 RNA promoter drives strong and ubiquitous GFP expression in transgenic *Xenopus* tadpoles [17]. Thus, we selected a 19-mer sequence optimal for HSF1 knockdown and placed the corresponding inverted repeat under the control of the human H1 RNA promoter. To allow for future identification of transgenic tadpoles, the H1-HSF1-sh1 cassette was linked in tandem with a muscle-specific GFP reporter.

**Figure 4:**
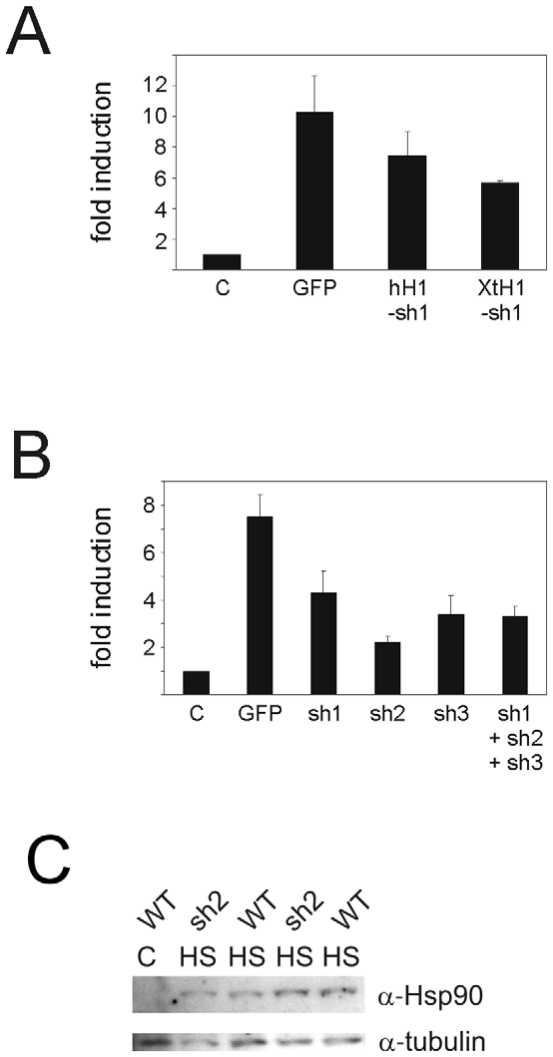
The effect of XHSF1 shRNAs on the HSR. (**A,B**) Reporter gene analysis of (**A**) the effect of HSF1 shRNAs expressed from the human or *Xenopus* H1 promoter, and (**B**) the effect of different shRNAs expressed from the *Xenopus* H1 promoter, on the HSR. A6 cells were transfected with mixtures of an Hsp70-luciferase reporter, a CMV-β-galactosidase reporter and the indicated plasmids. Relative luciferase activities and -fold induction were determined as described in the legend to fig. 2C. The results are the average of three independent transfections (standard deviations are indicated by error bars). (**B**) Western blot analysis of Hsp90 levels in tadpoles carrying the indicated transgenes. Lysates were made from whole tadpoles before heat shock (C) or after a 1 h heat shock at 33°C followed by 6 h recovery at room temperature (HS). The lysates were subjected to SDS-PAGE and western blot analysis using anti-Hsp90 and anti-tubulin antibodies.

To get a first impression of the inhibitory potential of the humH1-HSF1-sh1 plasmid, we tested its effect on the Hsp70-luciferase reporter in A6 cells. The humH1-HSF1-sh1 plasmid inhibited the heat shock induced activity of the Hsp70 promoter by only 27% (Fig. 4A). We reasoned that, in *Xenopus* tadpoles, the human H1 promoter may not be recognized by the proper RNA polymerase (polymerase II rather than III), which would result in shRNAs with long single-stranded extensions that do not induce RNAi. This would also explain why GFP expression from this promoter is so efficient [17], despite the fact that RNAs transcribed from polymerase III promoters are not usually capped or polyadenylated. Thus, we isolated an H1 (RNAse P) promoter from *Xenopus tropicalis* and expressed the HSF1-sh1 from this promoter. XtH1 promoter-driven HSF1-sh1 reduced the heat shock induced activity of a co-transfected Hsp70-luciferase reporter by almost 50% (Fig. 4A). Similar analyses of HSF1-sh2 and HSF1-sh3, which are directed against other parts of the HSF1 mRNA, resulted in, respectively, 71% and 55% inhibition of the HSR (Fig. 4B).

Since HSF1-sh2 had the strongest inhibitory activity in our *in vitro* system, this inverted repeat was used to generate transgenic tadpoles. Tadpoles carrying the HSF1-sh2 transgene were identified by muscle-specific expression of the GFP reporter. GFP-positive tadpoles developed normally and were equally resistant to sub lethal heat shock as non-transgenic tadpoles (data not shown). Western blot analysis indicated that heat shock mediated induction of endogenous Hsp90 expression was not inhibited in GFP positive tadpoles (Fig. 4C). Thus, despite its inhibitory activity on the HSR in A6 cells, HSF1-sh2 was not effective as an inhibitor of the HSR in tadpoles.

### Dominant-negative HSF1 mutant is embryonic lethal and not detectable in transgenic brain

**Figure 5:**
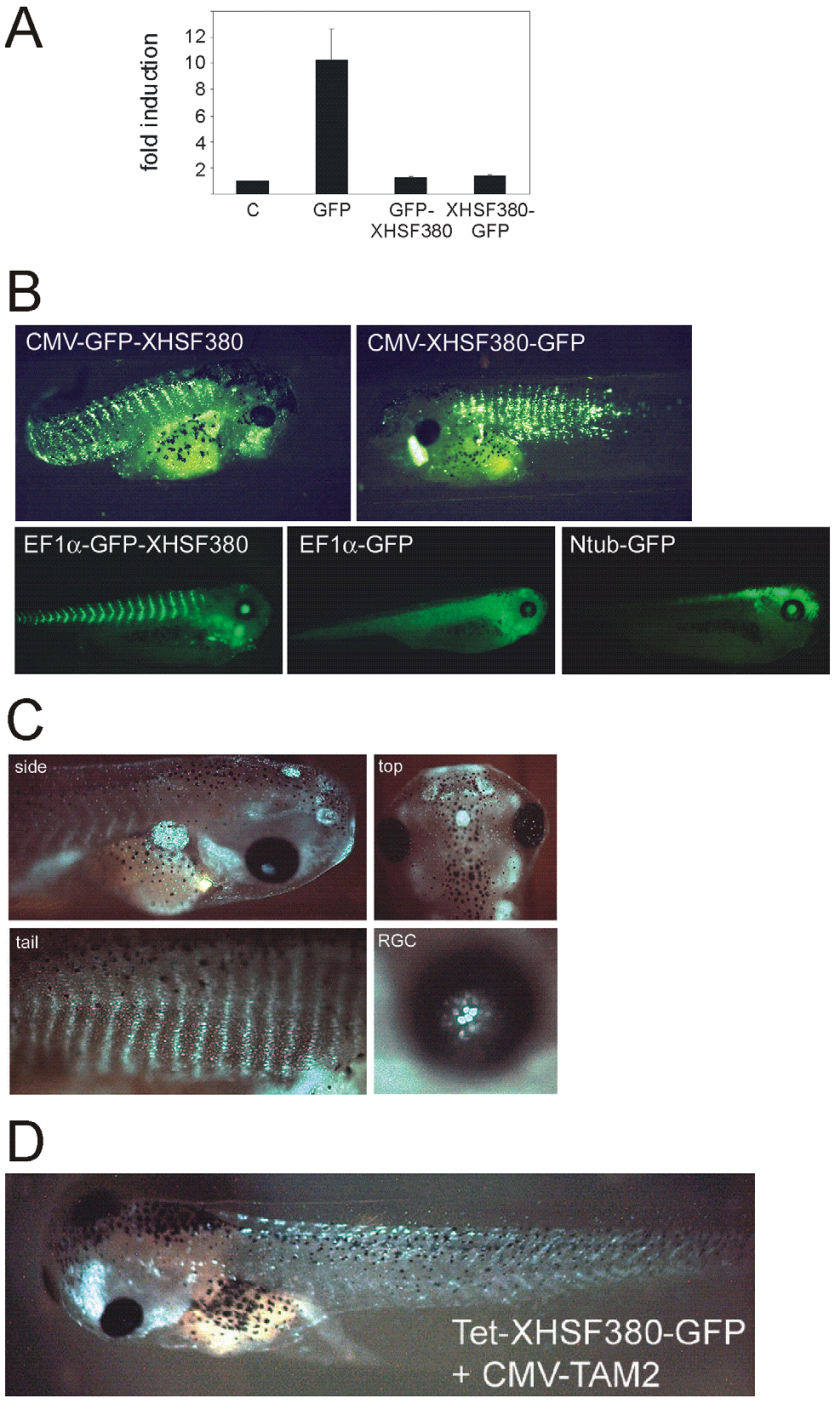
Transgene-driven expression of dominant-negative HSF1 in *Xenopus* larvae. (**A**) Reporter gene analysis of the effect of GFP-XHSF380 or XHSF380-GFP on the HSR. A6 cells were transfected with mixtures of an Hsp70-luciferase reporter, a CMV-β-galactosidase reporter and the indicated plasmids. Relative luciferase activities and -fold induction were determined as described in the legend to fig. 2C. The results are the average of three independent transfections (standard deviations are indicated by error bars). (**B**) Fluorescence microscope analysis of GFP-tagged XHSF380 constitutively expressed from either the CMV or the EF1α promoter in transgenic *Xenopus* larvae. Larvae with high constitutive expression of GFP-tagged XHSF380 develop poorly and never reach the feeding tadpole stage. Also shown are transgenic *Xenopus* larvae showing strong neuronal expression of GFP when driven by the EF1α or the Ntub promoter. (**C**) Fluorescence microscope analysis of transgenic *Xenopus* larvae expressing full length GFP-tagged XHSF1 from the CMV promoter. Fluorescence is pronounced in kidney, epiphysis, nasal epithelium, olfactory lobes, gills, retinal ganglion cell layer (RGC) and tail muscle nuclei. (**D**) Fluorescence microscope analysis of doxycycline-induced expression of GFP-tagged XHSF380 expressed in transgenic *Xenopus* larvae. A mixture of TetO-XHSF380-GFP and CS2+rtTA2A-M2 (CMV-TAM2) cassettes was used to generate transgenic *Xenopus* larvae. The larvae were allowed to develop in the absence of doxycycline until the feeding tadpole stage. GFP-negative larvae were exposed to doxycycline for 20 h and then monitored for GFP fluorescence.

Dominant-negative mutant versions of HSF1 have been successfully used by others to inhibit the HSR in mammalian systems (reviewed by [19]). Therefore, we tried to inhibit the HSR in tadpoles via transgene-mediated expression of dominant-negative HSF1. The first 380 codons of the XHSF1 open reading frame (XHSF380), thus lacking the code for the C-terminal trans activation domain, were PCR amplified from adult *Xenopus* brain cDNA. The XHSF380 cDNA was cloned upstream or downstream of the GFP code and the effect of expression of the GFP-XHSF380 fusion protein on the HSR was first tested via reporter gene analysis of the Hsp70 promoter in transiently transfected A6 cells. Heat shock mediated induction of Hsp70-Luc was completely inhibited by co-expression of GFP-XHSF380 or XHSF380-GFP, indicating that over expression of XHSF380 is a powerful way to inhibit the HSR (Fig. 5A).

We next used CMV-GFP-XHSF380 and CMV-XHSF380-GFP cassettes to generate transgenic tadpoles. GFP-positive larvae showed clear nuclear fluorescence (Fig. 5B), which is in accordance with an earlier report that *Xenopus* HSF1 is a nuclear protein even in the absence of heat stress [20]. Surprisingly, CMV promoter-driven expression of GFP-tagged XHSF380 was mainly restricted to skeletal muscle fibers and undetectable in brain and spinal cord (Fig. 5B), despite the fact that the CMV promoter is also highly active in neuronal tissue (data not shown). GFP-positive larvae (n = 40, Table 1) developed poorly, remained small, had a curved tail and died before reaching the feeding tadpole stage (Fig. 5B). Embryonic lethality could be due to the high expression level from the strong CMV promoter; however, replacing the CMV promoter with the weaker EF1α promoter, did not prevent the lethal phenotype. Again, GFP fluorescence was not detectable in neuronal tissues (data not shown), although expression of GFP-XHSBP1 from the EF1α promoter resulted in strong fluorescence in brain and spinal cord (Fig. 5B).

**Table 1:** Expression of XHSF380-GFP, GFP-XHSF380 or GFP from ubiquitous and neuron-specific promoters in transgenic *Xenopus* larvae.

Transgene	total number of larvae	number of GFP positive larvae	phenotype of GFP positive larvae
EF1α-GFP	75	8	normal
CMV-XHSF380-GFP	280	28	lethal
CMV-GFP-XHSF380	290	12	lethal
EF1α-GFP-XHSF380	255	13	lethal
Ntub-GFP	160	16	normal
Ntub-XHSF380-GFP	155	0	N.A.
Ntub-GFP-XHSF380	145	0	N.A.
CMV-GFP-XHSF1	225	6	normal

In a final effort to target expression of XHSF380 specifically to the brain, the CMV promoter was replaced with the neuron-specific β-tubulin (Ntub) promoter. Injections with Ntub-GFP-XHSF380 or Ntub-XHSF380-GFP cassettes resulted in ∼300 normal larvae without detectable GFP fluorescence, whereas ∼10% of the larvae derived from parallel injections with a Ntub-GFP cassette showed strong GFP fluorescence in brain and spinal cord (Fig. 5B, Table 1). We concluded that GFP-tagged XHSF380 cannot be stably expressed in *Xenopus* brain. The observation that both N-terminally and C-terminally GFP-tagged XHSF380 were undetectable in neuronal tissue suggested that this was due to tissue-specific degradation of the fusion protein or the corresponding mRNA, rather than inefficient translation of the transgene-derived mRNA.

To examine whether putative neuron-specific instability of XHSF380 was due to the absence of the C-terminal transcription activation domain, we also generated tadpoles expressing full length GFP-XHSF1 from the CMV promoter. Surprisingly, the expression pattern of GFP-XHSF1 was very similar to that of GFP-XHSF380. Fluorescence was pronounced in nuclei of muscle tissue, but undetectable in most parts of the brain, except for the epiphysis, the olfactory lobes and the retinal ganglion cells (Fig. 5C). In conclusion, the low neuronal expression level of transgene-derived XHSF380 is an intrinsic property of the *Xenopus* HSF1 protein and not a direct result of deletion of the transcription activation domain.

Since expressing XHSF380 was the most effective strategy to inhibit the HSR in *Xenopus* A6 cells, we tried to circumvent the embryonic lethality by suppressing XHSF380 expression during embryogenesis. The Tet-on system has been successfully used to regulate the expression of GFP and thyroid hormone receptor in *Xenopus* tadpoles [21]. GFP-XHSF380 and XHSF380-GFP cassettes under the control of a Tet-responsive CMV promoter were mixed with a Tet-activator expression cassette and used to generate transgenic larvae. Embryos were allowed to develop in the absence of doxycyclin. Subsequently, all normal GFP-negative tadpoles (n∼500) were exposed to doxycyclin to induce expression of GFP-tagged XHSF380. After 20 h doxycyclin treatment, ∼1% of the tadpoles showed nuclear GFP fluorescence (Fig. 5D), predominantly in muscle and again not in the nervous system, in spite of the fact that the TetO system has been shown to drive strong neuronal expression [21]. Further tadpole development in the presence of doxycyclin remained normal, indicating that the lethal effect of XHSF380 is stage-specific and that it can be circumvented by delaying the expression until after embryogenesis.

## Discussion

To mimic the aging-related decline of the HSR in a *Xenopus* tadpole model system, we tried to inhibit the activity of HSF1 using three different transgene-mediated strategies. XHSBP1 did not inhibit the HSR significantly, neither in A6 cells nor in transgenic tadpoles. Both untagged and GFP-tagged XHSBP1 were poor inhibitors, indicating that the absence of biological activity did not result from poor import into the nucleus due to the GFP tag. Perhaps, the expression level of XHSBP1 was insufficient to exert its inhibitory effect on HSF1, although the GFP fluorescence signal and western blot analysis suggested that the transgene was expressed at a very high level throughout the tadpole. The inhibitory effect of HSBP1 was described in a single study, wherein hemagglutinin-tagged human HSBP1 was shown to inhibit the HSR in COS7 cells and the *C. elegans* orthologue was shown to inhibit the HSR in nematodes *in vivo* [14]. The discrepancy between the published data and our results could be caused by species-specific differences in the biological activity of HSBP1, although its strong sequence conservation, especially in the hydrophobic core, suggests a conserved function. As our efforts to inhibit the HSR in human HeLa cells using human HSBP1 were also unsuccessful (unpublished results), the proposed biological function of HSBP1 may have to be reconsidered.

Stable transgene-driven RNAi directed against XHSF1 mRNA was not an effective means to inhibit the HSR in tadpoles. Earlier, it was shown that expression of exogenous GFP can be effectively inhibited by long GFP dsRNAs expressed from RNA polymerase II promoters [17] or GFP shRNAs expressed from the U6 RNA polymerase III promoter [18]. Here, we showed that XHSF1 shRNAs expressed from the *Xenopus tropicalis* H1 RNA promoter (RNA polymerase III promoter) inhibited the HSR by more than 70% in A6 cells. This implies that the RNAi-mediated inhibition of XHSF1 expression in A6 cells is highly efficient. Mice that are heterozygous for a null mutation in the HSF1 gene, still display a 100% HSR, whereas the HSR is completely abolished in homozygous null mutant mice [2]. There are several reasons why the XHSF1 shRNA might not be effective in transgenic tadpoles, despite its potent inhibitory activity in A6 cells. The heterogeneous genetic background of the parental frogs combined with the pseudotetraploid nature of their genome may result in mismatches between the shRNA sequence and the endogenous XHSF1 sequences, which would negatively influence the RNAi effect. Essential components of the RNAi machinery, such as the Dicer enzyme, may be present at suboptimal levels in tadpoles compared with A6 cells. In addition, activation of the RNAi machinery may require a threshold level of shRNAs that is reached in transiently transfected A6 cells, but not in stably transgenic tadpoles.

The dominant-negative XHSF380 mutant completely abolished the HSR in A6 cells. In tadpoles, its expression from a ubiquitously active promoter resulted in embryonic lethality, indicating that HSF1 signaling is required for normal *Xenopus* development. Surprisingly, HSF1 null mice also show abnormalities that are not directly related to stress, such as defects in female and male germ cells, placenta, and central nervous system [1]–[3], [22]–[24]. Together with our *Xenopus* data, these results indicate that HSF1 also functions under normal physiological conditions. The basal expression levels of several genes are reduced in specific tissues and embryonic fibroblast derived from HSF1 null mutant mice compared with wild type mice [25], [26]. Whether reduced expression of orthologous genes causes the embryonic lethality observed in tadpoles expressing XHSF380 remains to be determined. HSF1 target genes are expressed during early *Xenopus* development [27]. Embryonic lethality could be successfully circumvented by delaying the expression of XHSF380 until the feeding tadpole stage and a stable *Xenopus* line carrying a tetracyclin-inducible XHSF380 transgene may provide a useful model system to study the *in vivo* effects of a crippled HSR.

Surprisingly, GFP-tagged XHSF380 was not detectable in neuronal tissues, even when a neuron-specific promoter was used to specifically target expression to the brain. Expression from the ubiquitously active CMV or EF1α promoter resulted in clear GFP fluorescence in the nuclei of skeletal muscle fibers, indicating that the GFP-XHSF380 cassettes were properly transcribed and translated in non-neuronal tissue. Furthermore, parallel injections with an Ntub-GFP transgene resulted in high neuron-specific GFP fluorescence, indicating that the Ntub promoter functions properly (Fig. 5B). Thus, the absence of detectable GFP-tagged XHSF380 in transgenic brain and spinal cord most likely results from instability of the mRNA or protein. Full length GFP-XHSF1 expressed from the CMV promoter was also undetectable in most parts of the brain, but clearly visible in e.g. muscle and pronephros, again indicating that it is an intrinsic property of HSF1 or its coding sequence that causes the low neuronal expression. Similarly, a dominant positive HSF1 was not expressed in the brain of transgenic mice, in spite of the use of an ubiquitous promoter [7], [28]. Our data thus suggest the intriguing possibility that the low HSR in neuronal tissue [6] is due to tissue-specific differences in the half-life of HSF1.

## Materials and Methods

### Ethics statement

Animal experiments were carried out in accordance with the European Communities Council Directive 86/609/EEC for animal welfare, and were approved by the Radboud University Animal Experimentation Committee (permit TRC 99/15072 to generate and house transgenic *Xenopus*).

### Animal care

Female South-African claw-toed frogs (*Xenopus laevis*) were obtained from *Xenopus* Express (Cape Town, South-Africa) and kept in water tanks at 18°C at the Central Animal Facility of the Radboud University Nijmegen. Ovulation was induced by injection of 500 units hCG (Pregnyl; Organon) into the dorsal lymph sac.

### DNA constructs

Oligonucleotides that were used to generate recombinant DNA constructs are listed in the on line supplementary information (Table S1). New vectors were constructed to express proteins with N- or C-terminally fused GFP in *Xenopus* A6 cells and tadpoles. The chimeric enhancer/promoter of the *Xenopus* elongation factor-1α (EF1α) gene was PCR amplified from pEF-GFP3 (kindly donated by Dr. Paul Krieg; [29]) using the EF1a primer set, cut with *Sal*I and *Hin*dIII and used to replace the CMV promoter of the *Xenopus* vector pCS2+ [30], resulting in pEF2+. The code for GFPdelAUG was PCR amplified from pIRES2-EGFP (Clontech) using the GFPdelAUG primer set, and cloned into the *Bam*HI and *Xba*I sites of pEF2+, yielding pEF-GFPdelAUG. Similarly, the code for GFPdelSTOP was amplified from pIRES2-EGFP using the GFPdelSTOP primer set, and cloned into the *Bam*HI and *Xho*I sites of pEF2+, yielding pEF-GFPdelSTOP. Bicistronic expression vectors, based on viral 2A peptides, were generated as follows: pEF-GFP-T2A-delSTOP was made by annealing the T2A primer set, and cloning the double stranded oligo into the *Eco*RI and *Bgl*II sites of pEF-GFPdelSTOP. pEF-F2A-delAUG-GFP was made by annealing the F2A primer set, and cloning the double stranded oligo into the *Eco*RI and *Xho*I sites of pEF-GFPdelAUG.

Adult *Xenopus laevis* brain mRNA was used as a source for XHSBP1, XHSF and XHSF380 cDNA. Total RNA was isolated using the RNeasy kit (Qiagen) and copied into cDNA using the Marathon kit (Clontech). The 243-bp XHSBP1 cDNA was PCR amplified from the *Xenopus* brain cDNA library using the XHSBP1 primer set, and cloned into the *Eco*RI and *Xho*I sites of pEF-GFPdelSTOP, yielding pEF-GFP-XHSBP1. To express native protein, the XHSBP1 cDNA was also cloned into the *Eco*RI and *Xho*I sites of pEF-GFP-T2A-delSTOP, resulting in pEF-GFP-T2A-XHSBP1. The 1143-bp XHSF380 cDNA was PCR amplified from the *Xenopus* brain cDNA library using the XHSF380-C primer set, corresponding to GenBank sequence BC087308 from the NIH *Xenopus* initiative [31]. XHSF380 was cloned into the *Eco*RI and *Xho*I sites of pEF-GFPdelSTOP, yielding pEF-GFP-XHSF380. In parallel, the XHSF380 cDNA was PCR amplified using the XHSF380-N primer set, cut with *Bgl*II and *Eco*RI, and cloned into the *Bam*HI and *Eco*RI sites of pEF-GFPdelAUG, resulting in pEF-XHSF380-GFP. To drive strong ubiquitous expression of XHSF380-GFP and GFP-XHSF380, the EF1α promoter was replaced with the CMV promoter, resulting in pCMV-GFP-XHSF380 and pCMV-XHSF380-GFP. To drive neuron-specific expression, the EF1α promoter was replaced with the neural β-tubulin (Ntub) promoter, resulting in pNtub-GFP-XHSF380 and pNtub-XHSF380-GFP. Inducible expression constructs were generated by PCR amplifying the TetO promoter from pCS2+[tetO]::GFP using the TetO primer set, cutting the PCR fragment with *Sal*I and *Hin*dIII and replacing the EF1α promoter with the TetO promoter, resulting in pTetO-GFP-XHSF380 and pTetO-XHSF380-GFP. The Tet-activator plasmid pCS2+rtTA2A-M2 and reporter plasmid pCS2+[tetO]::GFP were kindly donated by Dr. Biswajit Das [21]. Full length pCMV-GFP-XHSF1 was made by amplifying the 3′ part of the XHSF1 code from the *Xenopus* brain cDNA library using the XHSF1 primer set, cutting the PCR fragment with *Eco*RV and *Xho*I and replacing the truncated *EcoR*V-*Xho*I fragment of pCMV-GFP-XHSF380 with the full length fragment.

The bicistronic reporter construct pEF-GFP-T2A-DsRed2 was made by amplifying the code for DsRed2 from pDsRed2-N1 (Clontech) using the DsRed2-C primer set, cutting the PCR fragment with *Bam*HI and *Xho*I, and cloning it into the *Bgl*II and *Xho*I sites of pEF-GFP-T2AdelSTOP. Similarly, pEF-DsRed2-F2A-GFP was made by PCR amplifying the code for DsRed2delSTOP from pDsRed2-N1 using the DsRed2-N primer set, and cloning the PCR fragment into the *Bam*HI and *Eco*RI sites of pEF-F2A-delAUG-GFP. The Hsp70-luciferase reporter construct pHL was described earlier [32].

For the purpose of inducing stable transgene-driven RNAi, new vectors were constructed that drive the synthesis of short hairpin RNAs (shRNAs) from an H1 RNA promoter. The human H1 RNA promoter was PCR amplified from genomic DNA using the H1 primer set, cut with *Sal*I and *Hin*dIII and used to replace the CMV promoter of pCS2+, resulting in pH1. To facilitate identification of transgenic tadpoles, a Cac-GFP-tkpolyA cassette, driving expression of the GFP reporter protein from the muscle-specific cardiac actin (Cac) promoter, was cloned into the *Not*I site of pH1, resulting in pH1CG2+. The *Xenopus laevis* RNAse P RNA (accession number X56558) and human H1 RNA sequences were used in a BLAST search of the *Xenopus tropicalis* genome (http://genome.jgi-psf.org), resulting in the identification of multiple copies of the *Xenopus* H1 RNA gene. Based on the alignment of six intact copies of the gene, the XtH1 primer set was designed and used to PCR amplify a *Xenopus tropicalis* H1 RNA promoter (XtH1). The human H1 RNA promoter in the pH1CG2+ vector was replaced with the XtH1 promoter using *Sal*I and *Hin*dIII, resulting in the pXtH1CG2+ vector.

Target sequences for RNAi-mediated decay of XHSF1 mRNA were chosen according to http://www.promega.com/siRNADesigner (Promega) and https://rnaidesigner.invitrogen.com/rnaiexpress (Invitrogen). pH1CG2+XHSF-sh1 was made by annealing of the XHSF-sh1a primer set, and cloning the double stranded oligo into the *Bgl*II and *Hin*dIII sites of pH1CG(A)2+. pXtH1CG2+XHSF1-sh1, -sh2 and –sh3 were made by annealing the XHSF-sh1, -sh2, and -sh3 primer sets, respectively, and cloning the double stranded oligos into the *Bsp*EI and *Hin*dIII sites of pXtH1CG2+.

The control plasmid pEF-GFP was made as follows: GFP cDNA was excised from pIRES2-EGFP (BD Sciences-Clontech) using *Msc*I and *Not*I (filled-in), introduced into the *Eco*RV site of pBluescript SK-, then excised again using *Hin*dIII (filled) and *Eco*RI and introduced into the *Stu*I and *Eco*RI sites of pEF2+.

All plasmids were sequence verified.

### *Xenopus* transgenesis

All expression cassettes, consisting of promoter, cDNA and polyadenylation signal, were excised from their plasmid vector backbones using *Sal*I and *Not*I, separated by agarose gel electrophoresis, and recovered from agarose slices using the GFX gel band purification kit (Amersham). Transgenesis of *Xenopus laevis* was performed according to Kroll and Amaya [33], with modifications [34]. In summary: 250,000 sperm nuclei were mixed with ∼200 ng DNA fragment, incubated for 15 min at room temperature and diluted in 500 µl sperm dilution buffer (250 mM sucrose, 75 mM KCl, 0.5 mM spermidine trihydrochloride, 0.2 mM spermidine tetrahydrochloride, 5 mM MgCl2, pH 7.4). Eggs were dejellied in 2% cystein/1×MMR (1×MMR: 0.1 M NaCl, 0.02 M KCl, 0.01 M MgCl_2_, 0.015 M CaCl_2_ en 0.5 M HEPES pH 7.5), transferred to 6% Ficoll/0.4×MMR and injected with 10 nl of the diluted nuclei/DNA mixture at 17°C. At the 4-cell stage, the embryos were transferred to 6% Ficoll/0.1×MMR and incubated overnight at 17°C. At the gastrula stage, the embryos were transferred to 0.1×MMR and incubated at 22°C. GFP-positive tadpoles were photographed using a MZ FLIII fluorescence stereomicroscope provided with a DC200 camera (Leica microsystems, Switzerland).

### Western blot analysis

Tadpoles or A6 cell pellets were snap frozen in liquid nitrogen and homogenized in 100 µl SDS-PAGE sample mix. Protein samples (25 µl) were separated in 12% polyacrylamide gels and transferred to nitrocellulose transfer membrane (Protran, Schleicher and Schuell) using a Biorad Mini-PROTEAN II Electrophoresis cell according to the manufacturer's instructions (Biorad). For western blot analysis, monoclonal α-GFP antibody (Cat. no. 632375; Clontech) was used at a 1∶5000 dilution. Monoclonal α-tubulin antibody (kindly donated by Mr. Huib Croes, Dept. of Cell Biology, NCMLS, Radboud University Nijmegen, The Netherlands) was used at a 1∶1000 dilution. Monoclonal anti-Hsp90 antibody (610418, BD Biosciences) was used at a 1∶1000 dilution. Proteins were visualized using the SuperSignal West Pico Chemiluminescent Substrate kit (Pierce).

### Tissue culture, transfections, and reporter gene assays

*Xenopus* A6 kidney epithelial cells (ATCC CCL-1020) were kindly donated by Ms. Stieneke van den Brink from the Hubrecht laboratory (Utrecht, the Netherlands). The cells were cultured at room temperature (∼25°C) in 70% Leibovitz medium supplemented with 10% fetal calf serum (FCS) and 25 mM Hepes pH 7.2. Transient transfection was performed using Fugene-6 (Roche) according to the manufacturer's instructions. Cells were seeded on six-well plates (2.5×10^5^ per well) and on the next day transfected with ∼1 µg plasmid per well in serum-free medium. The following day, the transfection mix was replaced with medium supplemented with FCS. For GFP and DsRed fluorescence analysis, cells were cultured on cover slips and fixed in 4% paraformaldehyde at 24 h after transfection. Fluorescence was monitored with a Leica DM RA microscope (Leica Microsystems), coupled to a Cohu high performance CCD camera. For luciferase assays, cells were transfected with a mixture of 100 ng pCMV-β-galactosidase, 200 ng pHL, and 800 ng of the plasmid indicated in the figures. At 48 h after transfection, cells were either left at room temperature (control) or incubated at 33°C for 1 hour (heat shock). After 6 h recovery at room temperature, cells were lysed in 200 µl reporter lysis mix (2.5 mM, 0.05% Tween 20, 0.05% Tween 80) for 10 min. For the β-galactosidase assay, 40 µl cell lysate was mixed with 100 µl phosphate solution [100 mM Na-phosphate pH 8.2, 10 mM MgCl_2_, 1% Galacton-Plus (Tropix)]. After 30 min incubation at room temperature, 150 µl accelerator II (Tropix) was added and luminescence was measured with the Lumat LB 9507 tube luminometer (Berthold technologies). For the luciferase assay, 40 µl cell lysate was mixed with 50 µl luciferin solution and luminescence was again measured with the Lumat luminometer. All reporter gene assays were performed *in triplo*.

## Supporting Information

Table S1 (0.05 MB DOC)
