# Supplementary material for: Manipulating Heat Shock Factor-1 in Xenopus Tadpoles: Neuronal Tissues Are Refractory to Exogenous Expression
Source: PLoS One. 2010 Apr 13;5(4):e10158. doi: 10.1371/journal.pone.0010158 (PMC2854154; doi:10.1371/journal.pone.0010158)
Supplement: Table S1 — (0.05 MB DOC) [file pone.0010158.s001.doc]

Supplementary table 1: Oligonucleotides that were used to generate recombinant DNA constructs

| name | sequence (5’ -> 3’) |
| --- | --- |
| EF1a-for | agctgtcgaccagggggatcatctaatcaag |
| EF1a-rev | agctaagcttcagctagaactcgccgcaga |
| GFPdelAUG-for | agctggatccggaattcggatatccatcgattctcgaggtgagcaagggcgaggagc |
| GFPdelAUG-rev | agcttctagattacttgtacagctcgtccatgc |
| GFPdelSTOP-for | agctggatccacaaccatggtgagcaa |
| GFPdelSTOP-rev | agctctcgagtgatatcgagatcttgaattccttgtacagctcgtccatgcc |
| T2A-up | aattgggatctggcgagggcagaggaagtcttctgacatgcggtgacgtggaggagaatcccggccctgaattcaa |
| T2A-low | gatcttgaattcagggccgggattctcctccacgtcaccgcatgtcagaagacttcctctgccctcgccagatccc |
| F2A-up | aattcgtgaaacagactttgaattttgaccttctcaagttggcgggagacgtggagtccaacccagggcccc |
| F2A-low | tcgaggggccctgggttggactccacgtctcccgccaacttgagaaggtcaaaattcaaagtctgtttcacg |
| XHSBP1-for | agctgaattcaccatgtcggagacagaccccaag |
| XHSBP1-rev | agctctcgagctattttkgcggcggckgctgc |
| XHSF380-C-for | agctgaattcaccatggacccccacgggacttgtg |
| XHSF380-C-rev | agctctcgagtcagcccggcactgaggaacaggag |
| XHSF380-N-for | agctagatctaccatggacccccacgggac |
| XHSF380-N-rev | agctgaattcgcccggcactgaggaacagg |
| TetO-for | cgctattacgccagtcgactttaccactccctatcag |
| TetO-rev | agctaagcttccgcggaggctg |
| XHSF1-for | gcctggtgaaacctgaaagagatg |
| XHSF1-rev | agctctcgagctaggagatgctggagcctgctg |
| DsRed2-C-for | agctggatcctcgccaccatggcctcctcc |
| DsRed2-C-rev | agctctcgagctacaggaacaggtggtggcg |
| DsRed2-N-for | agctggatcctcgccaccatggcctcctcc |
| DsRed2-N-rev | agctgaattccaggaacaggtggtggcg |
| H1-for | catggtcgacgaacgctgacgtcatcaacccg |
| H1-rev | gcaagcttagatctgtggtctcatacagaacttataagattccc |
| XtH1-for | agctgtcgacccagcagctcataactgaataagc |
| XtH1-rev | agctaagcttattccggacttcatagtatcttatatatatcccc |
| XHSF1-sh1a-up | gatccccgaggttcttccaaagtattcttcctgtcaaatactttggaagaacctctttttc |
| XHSF1-sh1a-low | agctgaaaaagaggttcttccaaagtatttgacaggaagaatactttggaagaacctcggg |
| XHSF1-sh1-up | ccggagtgaggttcttccaaagtattcttcctgtcaaatactttggaagaacctctttttc |
| XHSF1-sh1-low | agctgaaaaagaggttcttccaaagtatttgacaggaagaatactttggaagaacctcact |
| XHSF1-sh2-up | ccggagtgtacctgctttccttgccacttcctgtcatggcaaggaaagcaggtactttttc |
| XHSF1-sh2-low | agctgaaaaagtacctgctttccttgccatgacaggaagtggcaaggaaagcaggtacact |
| XHSF1-sh3-up | ccggagtgttgatatcagatgtacagcttcctgtcactgtacatctgatatcaactttttc |
| XHSF1-sh3-low | agctgaaaaagttgatatcagatgtacagtgacaggaagctgtacatctgatatcaacact |
